# Activation of Functional α7-Containing nAChRs in Hippocampal CA1 Pyramidal Neurons by Physiological Levels of Choline in the Presence of PNU-120596

**DOI:** 10.1371/journal.pone.0013964

**Published:** 2010-11-12

**Authors:** Bopanna I. Kalappa, Alexander G. Gusev, Victor V. Uteshev

**Affiliations:** Department of Pharmacology, Southern Illinois University School of Medicine, Springfield, Illinois, United States of America; Chiba University Center for Forensic Mental Health, Japan

## Abstract

**Background:**

The level of expression of functional α7-containing nicotinic acetylcholine receptors (nAChRs) in hippocampal CA1 pyramidal neurons is believed to be very low compared to hippocampal CA1 interneurons, and for many years this expression was largely overlooked. However, high densities of expression of functional α7-containing nAChRs in CA1 pyramidal neurons may not be necessary for triggering important cellular and network functions, especially if activation of α7-containing nAChRs occurs in the presence of positive allosteric modulators such as PNU-120596.

**Methodology/Principal Findings:**

An approach previously developed for α7-containing nAChRs expressed in tuberomammillary neurons was applied to investigate functional CA1 pyramidal α7-containing nAChRs using rat coronal hippocampal slices and patch-clamp electrophysiology. The majority (∼71%) of tested CA1 pyramidal neurons expressed low densities of functional α7-containing nAChRs as evidenced by small whole-cell responses to choline, a selective endogenous agonist of α7 nAChRs. These responses were potentiated by PNU-120596, a novel positive allosteric modulator of α7 nAChRs. The density of functional α7-containing nAChRs expressed in CA1 pyramidal neurons (and thus, the normalized net effect of activation, i.e., response net charge per unit of membrane capacitance per unit of time) was estimated to be ∼5% of the density observed in CA1 interneurons. The results of this study demonstrate that despite low levels of expression of functional pyramidal α7-containing nAChRs, physiological levels of choline (∼10 µM) are sufficient to activate these receptors and transiently depolarize and even excite CA1 pyramidal neurons in the presence of PNU-120596. The observed effects are possible because in the presence of 10 µM choline and 1–5 µM PNU-120596, a single opening of an individual pyramidal α7-containing nAChR ion channel appears to transiently depolarize (∼4 mV) the entire pyramidal neuron and occasionally trigger action potentials.

**Conclusions:**

1) The majority of hippocampal CA1 pyramidal neurons express functional α7-containing nAChRs. In the absence of PNU-120596, a positive allosteric modulator of α7 nAChRs, a lack of responsiveness of some hippocampal CA1 pyramidal neurons to focal application of 0.5–1 mM choline does not imply a lack of expression of functional α7-containing nAChRs in these neurons. Rather, it may indicate a lack of detection of α7-containing nAChR-mediated currents by patch-clamp electrophysiology. 2) PNU-120596 can serve as a powerful tool for detection and enhancement of responsiveness of low densities of functional α7-containing nAChRs such as those present in hippocampal CA1 pyramidal neurons. 3) In the presence of PNU-120596, physiological concentrations of choline activate functional CA1 pyramidal α7-containing nAChRs and produce step-like currents that cause repetitive step-like depolarizations, occasionally triggering bursts of action potentials in CA1 pyramidal neurons. Therefore, the results of this study suggest that in the presence of PNU-120596 and possibly other positive allosteric modulators, endogenous choline may persistently activate CA1 pyramidal α7-containing nAChRs, enhance the excitability of CA1 pyramidal neurons and thus act as a potent therapeutic agent with potential neuroprotective and cognition-enhancing properties.

## Introduction

The hippocampus is a brain region that supports essential cognitive functions and memory formation [1]. Hippocampal CA1 pyramidal neurons are important for learning, short-term memory and memory consolidation [2], [3], [4] and are particularly vulnerable to neurodegenerative processes associated with Alzheimer's disease, aging, brain trauma and cerebral ischemia [5], [6], [7], [8]. Moreover, the excitability of CA1 pyramidal neurons may positively correlate with cognitive performance, and has been shown to decline with age likely due to an age-dependent enhancement of inhibitory effects of Ca^2+^-dependent potassium conductances [9], [10], [11]. Therefore, therapeutic approaches that provide neuroprotection and restore excitability of hippocampal CA1 pyramidal neurons may benefit patients with various forms of dementia and brain trauma.

The hippocampus receives cholinergic inputs from the basal forebrain and expresses multiple pre- and postsynaptic nAChR subunits [12], [13]. Nicotine and other nicotinic agents including selective α7 nAChR agonists have been shown to provide neuroprotection and reduce cognitive decline associated with Alzheimer's disease, schizophrenia, brain trauma and aging [9], [14], [15], [16], [17], [18], [19], [20], [21], [22], [23], [24], [25], [26], [27], [28], [29], [30], [31]. Although the majority of hippocampal neurons express α7-containing (i.e., α7*) nAChR subunits [32], functional hippocampal α7* nAChRs are less common. In fact, for many years the expression of functional α7* nAChRs in hippocampal CA1 pyramidal neurons was largely overlooked. Although functional nAChRs have been detected on both excitatory (i.e., glutamatergic) and inhibitory (i.e., GABAergic) pre-synaptic terminals, postsynaptic functional α7* nAChRs were initially believed to be expressed largely on the inhibitory GABAergic interneurons and not on the principal pyramidal neurons [33], [34], [35], [36]. This position has recently been challenged by at least two studies where whole-cell responses of CA1–3 pyramidal neurons to ACh or nicotine were detected in electrophysiological and fluorescent Ca^2+^ imaging experiments in hippocampal slices [37], [38]; and activation of post-synaptic, likely dendritic, α7* nAChRs in CA1 pyramidal neurons supported long term potentiation [38]. A failure of early studies to consistently detect α7* nAChR-mediated responses in CA1 pyramidal neurons may be explained by the relatively low density of expression and a potential distal/dendritic location of functional pyramidal α7* nAChRs.

PNU-120596, a novel positive allosteric modulator of α7* nAChRs, increases the potency of nicotinic agonists [39], [40], [41], [42], [43] enhancing the responsiveness of functional α7* nAChRs and producing therapeutic benefits in animal models [40]. In rats, intravenous injections of 1 mg/kg PNU-120596 elevated the concentration of PNU-120596 in the cerebrospinal fluid to ∼1.5 µM [40]. This value falls near the EC_50_ for potentiating effects of PNU-120596 (EC_50_∼1.5 µM) [44], [45]. Concentrations slightly lower than the EC_50_ (i.e., 1 µM PNU-120596) have been shown to enhance the effects of sub-threshold concentrations of choline, a selective endogenous agonist of α7* nAChRs, allowing physiological levels of choline (i.e., 5–10 µM) to become effective in activation of native α7* nAChRs in the absence of exogenous nicotinic agents [43]. Although choline is found in the cerebrospinal fluid in *in vivo* preparations at concentrations near 5–10 µM [46], [47], [48], [49], [50], [51], [52], [53], isolated brain slices are likely choline-depleted and may contain only sub-µM levels of choline (V. Uteshev, R. Papke and L. Prokai, unpublished). Therefore, to model the effects of endogenous choline on CA1 pyramidal α7* nAChRs in hippocampal slices, physiological concentrations of choline (i.e., ∼10 µM) need to be added to artificial cerebrospinal fluid (ACSF).

Additional challenges for detection of whole-cell α7* nAChR-mediated responses arise from the extremely short mean open time (∼100 µs) of individual α7* nAChR ion channels [54]. Therefore, to produce a clearly detectable whole-cell current, activation of individual α7* nAChRs has to be well-synchronized which may be difficult to achieve in whole-cell experiments in brain slices. PNU-120596 has been shown to increase the mean open time of α7 nAChR ion channels without producing significant changes in ion channel selectivity, Ca^2+^ permeability and single channel conductance [40]. PNU-120596 does not activate native functional α7* nAChRs in the absence of nicotinic agonists [43], instead, it lowers the energy barrier and allows lower concentrations of nicotinic agonists to activate α7* nAChRs [55]. When administered alone intravenously, low concentrations of PNU-120596 (i.e., 0.1–1.5 µM) were effective in reversing the amphetamine-induced auditory gating deficit in rats [40] likely via potentiating the effects of endogenous choline on native α7* nAChRs.

The concentration of endogenous choline (i.e., 5–10 µM) in the cerebrospinal fluid (CSF) is too low to activate native α7* nAChRs [43], [56], especially in hippocampal CA1 pyramidal neurons where expression of α7* nAChRs appears limited [33], [34], [38]. Therefore, choline is not usually considered an effective therapeutic agent. However, recently this laboratory has proposed that in the presence of PNU-120596, 5–10 µM choline may become effective in producing a weak but persistent activation of α7* nAChRs in hypothalamic tuberomammillary (TM) neurons known to express high densities of functional α7* nAChRs [43]. In addition to producing a weak persistent activation, synergistic action of 1 µM PNU-120596 plus 5–10 µM choline generated whole-cell responses that were reminiscent of and postulated to be single α7* nAChR ion channel openings detectable in whole-cell patch-clamp configuration [43]. Since in the presence of PNU-120596, a single opening of α7* nAChR ion channel was sufficient for significant depolarization and even excitation of the entire TM neuron, high densities of expression of functional α7* nAChRs may not be necessary for a considerable enhancement of excitability of α7* nAChR-expressing neurons under similar conditions. The present study tests the hypothesis that the majority of hippocampal CA1 pyramidal neurons express functional α7* nAChRs although at relatively low densities compared to TM neurons or hippocampal CA1 interneurons. Secondly, this study demonstrates that in the presence of PNU-120596, physiological levels of choline produce a weak persistent activation of CA1 pyramidal α7* nAChRs and enhance excitability of hippocampal CA1 pyramidal neurons. This weak persistent activation of α7* nAChRs may be neuroprotective [9], [14], [16], [22], [57] and cognitively beneficial [15], [23], [25], [26], [27], [28], [29], [58].

## Materials and Methods

### Animals

Young adult male and female Sprague-Dawley rats (P18–30) were used in experiments. Hippocampal slices from male and female rats were found to be consistently similar and expressed functional α7* nAChRs with similar properties. However, this study did not specifically investigate gender dependence of functional α7* nAChRs in the hippocampus. Animal care was in accordance with the Guide for the Care and Use of Laboratory Animals (NIH 865-23, Bethesda, MD). Full details of the study were approved by the Animal Care and Use Committee of Southern Illinois University (Protocols 197-06-011 and 197-07-018).

### Preparation of brain slices

To exclude potential drug interference, general or local anesthetics were not used and animals were euthanized via a rapid decapitation. Brains were rapidly removed and transferred to an ice-cold sucrose-rich solution of the following composition (in mM): sucrose 250, KCl 3, NaH_2_PO_4_ 1.23, MgCl_2_ 5, CaCl_2_ 0.5, NaHCO_3_ 26, glucose 10 (pH 7.4), when bubbled with carbogen (95% O_2_ and 5% CO_2_). Three to four coronal whole-brain slices (260 µm thick) containing the hippocampus were cut in a sucrose-rich solution at 3°C using Vibratom-1000+ (Vibratom, St. Louis, MO). Slices were transferred to a temporary storage chamber where they were maintained for ∼30 min at 30°C in an oxygenated artificial cerebral-spinal fluid (ACSF) of the following composition (in mM): NaCl 125, KCl 3, NaH_2_PO_4_ 1.23, MgCl_2_ 1, CaCl_2_ 2, NaHCO_3_ 26, glucose 10 (pH 7.4), when bubbled with carbogen. Slices were then transferred back into the storage chamber and maintained at room temperature bubbled with carbogen. The majority of experiments were conducted within ∼6 hours after the tissue preparation. However, experiments occasionally lasted longer than expected and slices were incubated in carbogenated ACSF for up to 10 hours. Regardless of the duration of incubation, if slices and/or neurons showed signs of damage (e.g., slices lost their original shape, texture or color; or neurons became granulated, shapeless or borderless with clearly seen nuclei), then slices were discarded and a new tissue preparation was conducted or the work was postponed until the next day.

### Drugs

PNU-120596 (*N*-(5-Chloro-2,4-dimethoxyphenyl)-*N*'-(5-methyl-3-isoxazolyl)-urea) was purchased from Tocris Bioscience (Ellisville, MO). Gabazine (6-Imino-3-(4-methoxyphenyl)-1(6*H*)-pyridazinebutanoic acid hydrobromide), DNQX (6,7-dinitroquinoxaline-2,3-dione), AP-5-Na^+^ (DL-2-Amino-5-phosphonopentanoic acid sodium salt) and tetrodotoxin (TTX) were purchased from Ascent Scientific (Bristol, UK). Other chemicals were purchased from Sigma-Aldrich (St. Louis, MO). Choline-containing solutions were freshly made immediately prior to each experiment from a 1 M choline stock solution which was kept frozen at −20°C. Previous studies reported that, in heterologous systems, the EC_50_ for potentiating effects of PNU-120596 was near 1.5 µM [44], [45]. Therefore, in the present study, 2 µM and 5 µM PNU-120596 were tested. These concentrations of PNU-120596 may be therapeutically relevant because intravenous administration of 1 mg/kg PNU-120596 has been shown to elevate the concentration of PNU-120596 in the brains of rats to similar values (∼1.5 µM) producing therapeutically beneficial effects [40].

In the majority of experiments, ACSF contained the following antagonists to inhibit GABA_A_, AMPA, NMDA, muscarinic ACh and GABA_A_/glycine receptors, respectively: 20 µM gabazine, 15 µM DNQX, 50 µM AP-5, 10 µM atropine and 40 µM picrotoxin. In voltage-clamp experiments, 0.3 µM TTX was typically added to ACSF to inhibit voltage-gated sodium ion channels.

### Patch-clamp recordings

For patch-clamp experiments, slices were transferred to the recording chamber perfused with ACSF at a rate of 1 ml/min using a perfusion pump 2232 Microperpex S (LK.B, Upsalla, Sweden). Hippocampal CA1 pyramidal neurons and CA1 interneurons were selected under visual control using an infrared Olympus BX-51WI microscope (Olympus America Inc, Center Valley, PA). Electrophysiological patch-clamp recordings were made using a MultiClamp-700B amplifier equipped with Digidata-1440A A/D converter (Molecular Devices, Sunnyvale, CA). Data were filtered at 4–8 kHz, sampled at 20–50 kHz and stored on a personal computer for offline analysis. For recordings, patch pipettes were pulled using a Sutter P-97 horizontal puller (Sutter Instruments, Novato, CA). The pipette resistance was ∼4–6 MΩ when filled with the internal solution (see below). After formation of a stable gigaseal (>2 GΩ), the whole-cell configuration was established. Choline (0.5–1 mM) was applied via picospritzer pipettes (application pressure 5–8 psi, Parker Hannifin Instrumentation, Cleveland, OH, USA) identical to those used for patch-clamp recordings. The tip of application pipette was positioned ∼15 µm from the recorded neuron. PNU-120596 (1–5 µM) was applied to ACSF. Whole-cell recordings were conducted at room temperature. The membrane voltage in voltage-clamp experiments was -60 mV, unless otherwise specified. The extracellular solution was identical to ACSF which was used for the brain tissue preparation. The intracellular electrode solution contained (in mM): K-gluconate 140, NaCl 1, MgCl_2_ 2, Mg-ATP 2, Na-GTP 0.3, HEPES 10, KOH 0.42 (pH 7.38). To reduce the recording noise, in experiments where responses of α7* nAChRs in CA1 pyramidal neurons and CA1 interneurons were compared, a Cs-methanesulfonate-based internal solution was used with the following composition (in mM): CsMeSO_3_ 140, NaCl 6, MgCl_2_ 2, Mg-ATP 2, Na-GTP 0.3, HEPES 10, CsOH 0.3 (pH 7.38). Membrane voltages were not corrected for the liquid junction potentials: V_LJ_(K-gluconate) = 16.2 mV and V_LJ_(CsMeSO_3_) = 9.8 mV. When necessary, a GENIE Plus syringe pump (Kent Scientific, Torrington, CT) was used to add 0.3 µM tetrodotoxin (TTX, a Na^+^ ion channel antagonist) or 20 nM methyllycaconitine (MLA, a selective α7 nAChR antagonist) to the ACSF just before it entered the recording chamber. The final drug concentrations in the chamber were then calculated based on the known concentrations of drug stock solutions and adjustable rates of all pumps. The application solutions containing various concentrations of choline were prepared fresh daily.

### Analysis

The analysis of current-deviations and action potentials was conducted using Clampfit-10.1 software program. A threshold search event detection protocol (Clampfit-10.1) was used to evaluate the frequencies of step-like current deviations and action potentials. To measure current net charge, choline and PNU-120596 were added to ACSF for at least 20 min and step-like responses of hippocampal CA1 pyramidal neurons in rat brain slices were continuously recorded in voltage-clamp in 10 min intervals and analyzed offline. The experimental results are presented as the mean ± S.D., unless otherwise indicated. Curve fitting was done using ProStat analysis program (Poly Software International, Pearl River, NY).

## Results

**Figure 1:**
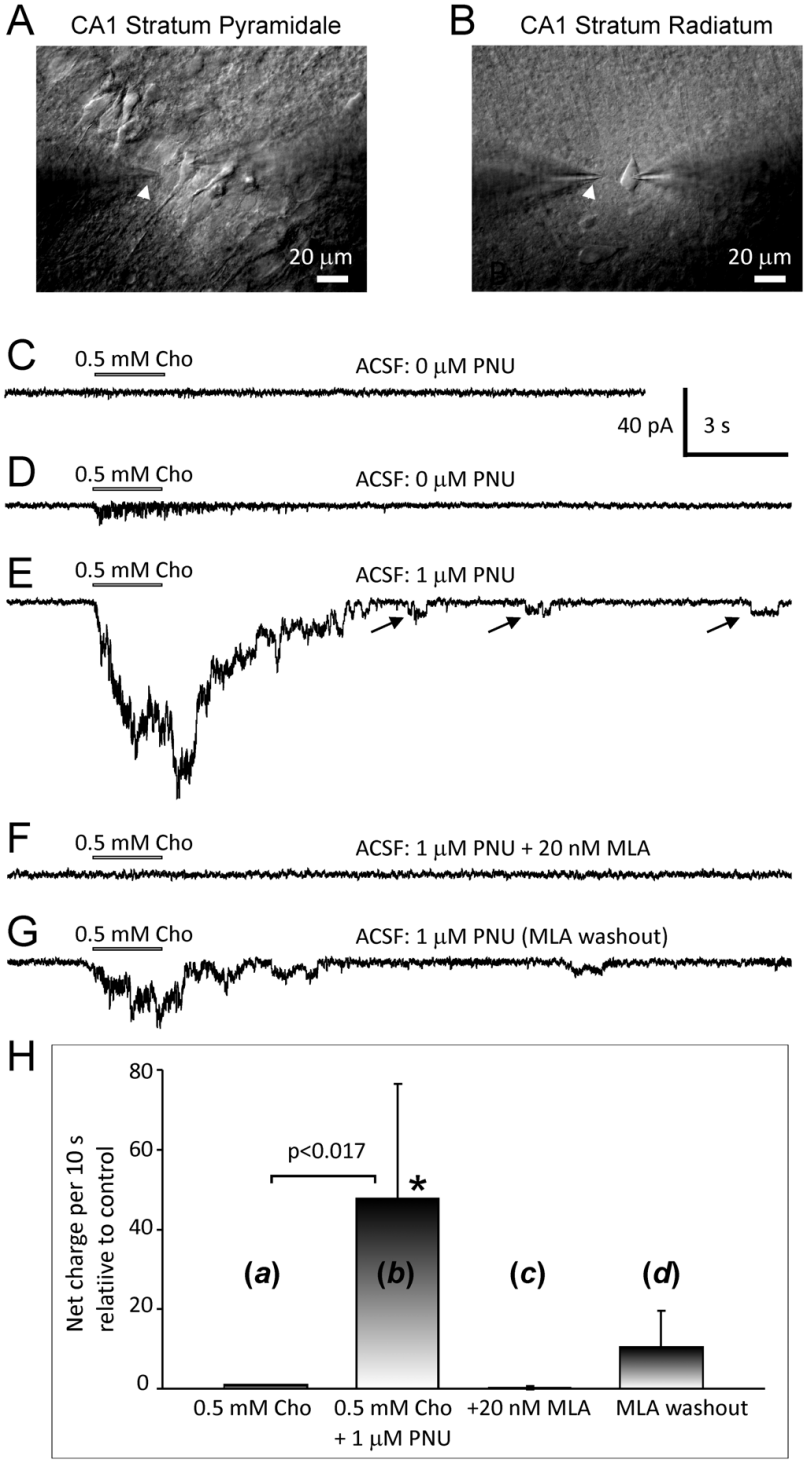
Responses of hippocampal CA1 pyramidal neurons to pressure-applied choline in the absence and presence of PNU-120596. In hippocampal slices, CA1 pyramidal neurons (A) can be easily distinguished from interneurons (B) based on their location and shape. White arrows indicate the positions of the tips of application pipettes (A–B). Usually, CA1 pyramidal neurons do not exhibit robust responsiveness to choline, a selective endogenous α7 nAChR agonist (C–D). Pressure application of 0.5–1 mM choline to pyramidal neurons elicited detectable whole-cell responses in 71% of voltage-clamp experiments. Addition of 1 µM PNU-120596 to ACSF considerably enhanced the responsiveness of CA1 pyramidal neurons to choline (E). These responses were completely and reversibly blocked by 20 nM methyllycaconitine (MLA) (F–G). In the presence of PNU-120596, step-like currents were routinely observed in whole-cell experiments (arrows, E). H) To quantify these effects, in four experiments with CA1 pyramidal neurons, 0.5 mM choline was pressure-applied 3–4 times in the absence of PNU-120596 (H, *a*); then 1 µM PNU-120596 was added to ACSF for at least 50 min and responses to pressure-applied 0.5 mM choline were recorded again (H, *b*); 20 nM MLA was then added to ACSF for ∼15 min resulting in a complete block of choline-evoked responses (H, *c*); and finally, MLA was removed from ACSF resulting in a partial reversal of responses after ∼50 min of MLA washout (H, *d*). Net charge of step-like currents was measured over the first 10 s after the beginning of choline puff. Responses of hippocampal CA1 pyramidal neurons to pressure-applied 0.5 mM choline in the absence (H, *a*) and presence (H, *b*) of 1 µM PNU-120596 were significantly different (p<0.017; n = 4). In each experiment, net charge values were normalized to the net charge of control responses (H, *a*).

Patch-clamp electrophysiological recordings were conducted using coronal hippocampal slices of young adult rats to test and confirm the hypothesis that in the presence of PNU-120596, physiological concentrations of choline (i.e., ∼10 µM) are effective in eliciting a weak but persistent activation of functional hippocampal CA1 pyramidal α7* nAChRs and that high densities of expression of these receptors are not required to cause significant effects on excitability of hippocampal CA1 pyramidal neurons. The majority of experiments were conducted in the absence of glutamatergic, gabaergic/glycinergic and muscarinic AChR-mediated inputs. A total of 321 hippocampal CA1 pyramidal neurons and 72 hippocampal interneurons were studied. The recorded neurons were identified by their morphology and location within the slice. Specifically, hippocampal CA1 pyramidal neurons had a distinct pyramidal shape (Figure 1A) and were located in the CA1 *Stratum Pyramidale* region of the hippocampus (Figure 1A). Typically, these neurons did not exhibit robust responsiveness to pressure-applied 0.5–1 mM choline in voltage-clamp whole-cell experiments (Figure 1C–D). By contrast, hippocampal CA1 interneurons had a more rounded shape (Figure 1B) and were located in the CA1 *Stratum Radiatum* region of the hippocampus (Figure 1B). Typically, these neurons responded to pressure-applied 0.5–1 mM choline with robust currents in voltage-clamp whole-cell experiments (see below and [33], [35]).

### Expression of native functional α7* nAChRs in hippocampal CA1 pyramidal neurons

In the initial set of whole-cell experiments, 0.5–1 mM choline, a selective endogenous agonist of α7 nAChRs, was focally administered to the recorded hippocampal CA1 pyramidal neurons via a picospritzer pipette identical to that used for patch-clamp recordings. In these experiments, the tip of application pipette was positioned within ∼15 µm from the recorded cell soma (white arrows, Figure 1A) and brief (1–2 s) puffs of choline were delivered every 2–3 min. Pressure-applied 0.5–1 mM choline (∼5 psi pressure) elicited either no responses (Figure 1C) or small whole-cell currents (<30 pA in amplitude, Figure 1D). In this study, pressure-applied 0.5–1 mM choline produced detectable responses (i.e., current peak/noise >0.5) in 52 out of 73 tested CA1 pyramidal neurons (∼71%) (e.g., Figure 1D). The remaining 21 neurons (∼29%) did not respond to 0.5–1 mM choline by clearly detectable current deviations (e.g., Figure 1C).

As expected, addition of 1–5 µM PNU-120596 to ACSF potentiated responses of CA1 pyramidal neurons to pressure-applied 0.5–1 mM choline (Figure 1E). These responses were completely and reversibly blocked by 20 nM methyllycaconitine (MLA; n = 11), a selective antagonist of α7 nAChRs (Figure 1F–G), supporting the involvement of functional CA1 pyramidal α7* nAChRs. In the presence of PNU-120596, some whole-cell current deviations were step-like (arrows, Figure 1E) and reminiscent of openings of individual ion channels as reported previously for hypothalamic tuberomammillary α7* nAChRs [43].

To quantify these effects an identical experimental protocol was used in four CA1 pyramidal neurons (*a-d*; Figure 1H): (*a*) 0.5 mM choline was pressure-applied 3–4 times in the absence of PNU-120596; (*b*) PNU-120596 (1 µM) was then added to ACSF for at least 50 min and responses to pressure-applied 0.5 mM choline were recorded again; (*c*) MLA (20 nM) was then applied to ACSF for ∼15 min resulting in a complete block of choline-evoked responses and (*d*) MLA was removed from ACSF resulting in a partial reversal of responses after ∼50 min of washout. Net charge of step-like currents over the first 10 s after the beginning of choline puff was measured for each of the four cells tested, the results were averaged and plotted (Figure 1H). Addition of 1 µM PNU-120596 to ACSF significantly enhanced responses of hippocampal CA1 pyramidal neurons to pressure-applied 0.5 mM choline (p<0.017; n = 4; Figure 1H, columns (*a,b*)). All net charge values (i.e., *b–d*) in each given experiment were normalized to net charge of the control response (i.e., *a*).

**Figure 2:**
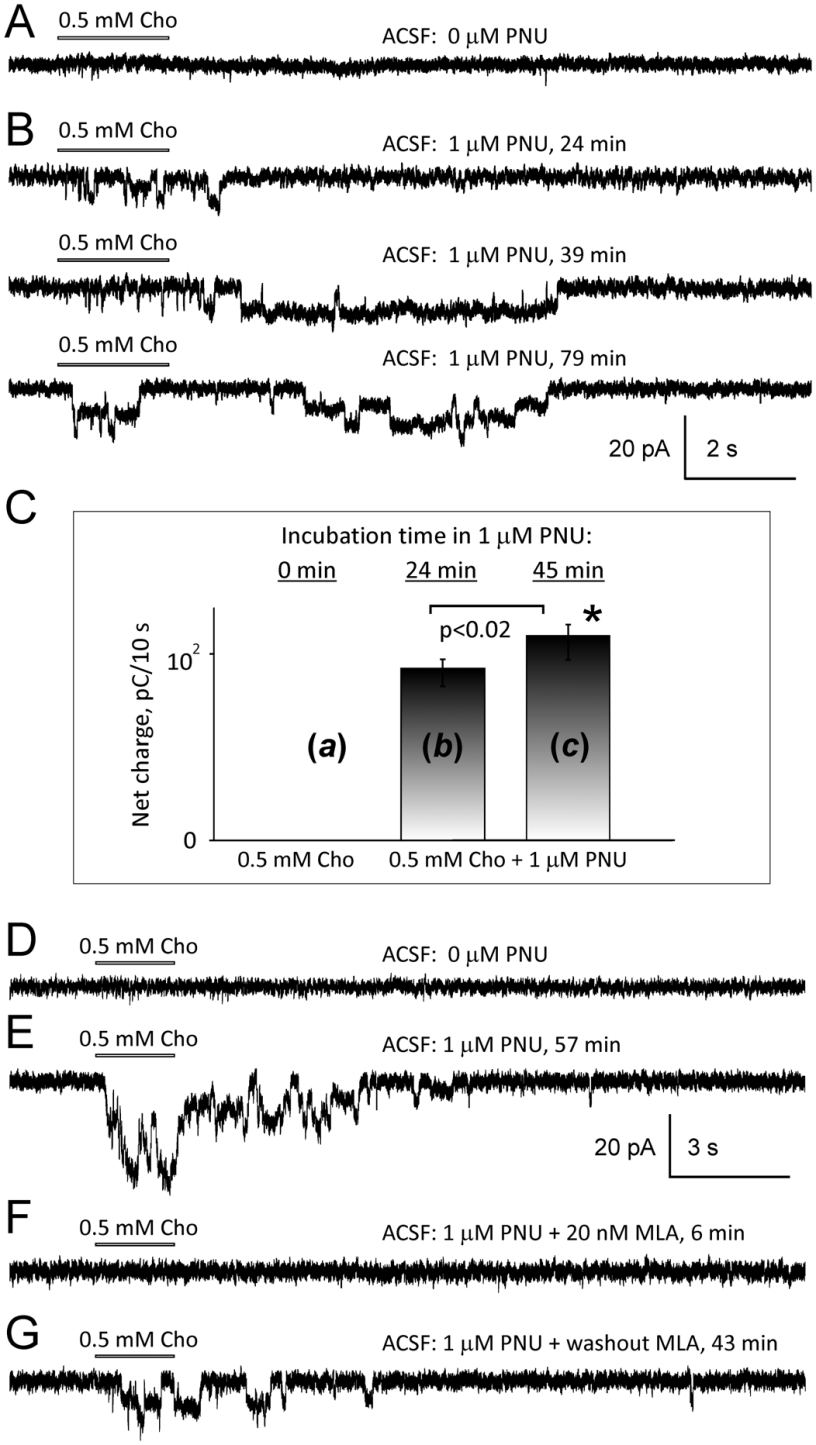
Improved detection of α7* nAChR-mediated currents in hippocampal CA1 pyramidal neurons in the presence of PNU-120596. In some patch-clamp experiments conducted in hippocampal slices, focal pressure administration of 0.5 mM choline to hippocampal CA1 pyramidal neurons did not elicit detectable whole-cell currents (A and D). Traces A and D were obtained from different CA1 pyramidal neurons. Trace D is the same as trace C in Figure 1. The same neurons began to respond to choline after addition of 1 µM PNU-120596 to ACSF (B and E). These responses were completely and reversibly blocked by 20 nM MLA (F–G) supporting the expression of functional α7* nAChRs. C) To quantify these effects, in six experiments in which in the absence of PNU-120596 hippocampal CA1 pyramidal neurons did not respond to pressure-applied 0.5 mM choline, addition of 1 µM PNU-120596 to ACSF resulted in robust responses. Current net charge over the first 10 s after the beginning of choline puff was measured for three experimental phases: (C, *a*) in the absence of PNU-120596; (C, *b*) ∼24 min after addition of PNU-120596 to ACSF; and (C, *c*) ∼45 min after addition of 1 µM PNU-120596 to ACSF. Net charge values measured at ∼24 min and ∼45 min after PNU-120596 administration were significantly different (p<0.02, n = 6) indicating time-dependence of PNU-120596 effects within the time range used in these experiments.

In some cases, a lack of responses of CA1 pyramidal neurons to focal administration of 0.5–1 mM choline (Figures 2A and 2D) may have reflected an inability of whole-cell patch-clamp recordings to detect α7* nAChR-mediated currents in hippocampal CA1 pyramidal neurons in slices rather than a lack of expression of functional α7* nAChRs in these neurons. To test this hypothesis, 1–5 µM PNU-120596 was added to ACSF in experiments where CA1 pyramidal neurons did not respond to 0.5–1 mM choline (Figures 2A and 2D). Indeed, 17 out of 18 tested CA1 pyramidal neurons that did not initially respond to pressure-applied choline (Figures 2A and 2D) began responding to choline after addition of PNU-120596 to ACSF (Figures 2B and 2E).

To quantify these effects, an identical experimental procedure was used in six experiments in which in the absence of PNU-120596 hippocampal CA1 pyramidal neurons did not respond to pressure-applied 0.5 mM choline (e.g., Figures 2A and 2D). Addition of 1 µM PNU-120596 to ACSF enhanced the responsiveness of α7* nAChRs expressed in these neurons resulting in step-like responses (e.g., Figures 2B and 2E). Current net charge over the first 10 s after the beginning of choline puff was measured and plotted for three experimental phases: (*a*) in the absence of PNU-120596; (*b*) ∼24 min after addition of PNU-120596 to ACSF; and (*c*) ∼45 min after addition of 1 µM PNU-120596 to ACSF (Figure 2C). Net charge values measured at ∼24 min and ∼45 min after PNU-120596 administration were significantly different (p<0.02, n = 6; Figure 2C). Potentiated responses were also completely and reversibly blocked by 20 nM MLA (n = 3; Figure 2E–G). These results demonstrated that a lack of responsiveness of CA1 pyramidal neurons to 0.5 mM choline does not necessarily reflect a lack of expression of functional α7* nAChRs.

The responses of hippocampal CA1 pyramidal α7* nAChRs to 0.5–1 mM choline were resistant to 0.3 µM TTX, 20 µM gabazine, 15 µM DNQX, 50 µM AP-5, 10 µM atropine and 40 µM picrotoxin which were routinely added to ACSF to inhibit Na^+^ voltage-gated ion channels, GABA_A_, AMPA, NMDA, muscarinic ACh and GABA_A_/glycine receptors, respectively.

These initial voltage-clamp experiments used focal pressure application of relatively high concentrations of choline (0.5–1 mM) to demonstrate that: 1) Hippocampal CA1 pyramidal neurons express functional α7* nAChRs as previously reported [37], [38]; and 2) A lack of responsiveness of some hippocampal CA1 pyramidal neurons to focal application of 0.5–1 mM choline in the absence of PNU-120596 does not imply a lack of expression of functional α7* nAChRs in these neurons. Rather, it may indicate a lack of detection of α7* nAChR-mediated currents by patch-clamp electrophysiology.

### Comparison of the net effects of activation of native functional α7* nAChRs in CA1 pyramidal neurons and CA1 interneurons

**Figure 3:**
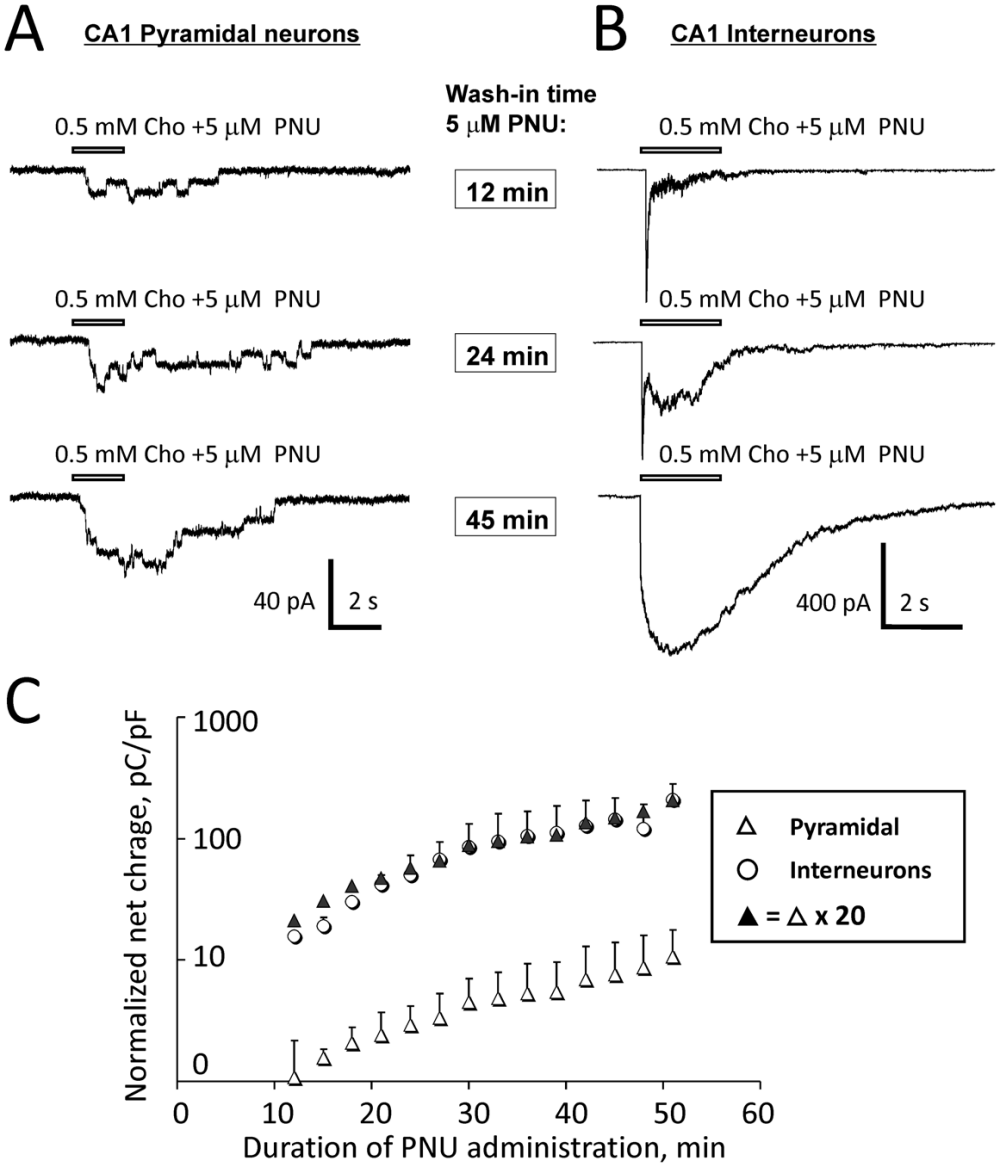
Comparison of the levels of expression of native functional α7* nAChRs in CA1 pyramidal neurons and CA1 interneurons. The densities of expression of functional α7* nAChRs in CA1 pyramidal neurons and CA1 interneurons were compared in voltage-clamp whole-cell experiments in hippocampal slices. PNU-120596 (5 µM) was added to ACSF to enhance the detectability of α7* nAChR-mediated responses. In the presence of 5 µM PNU-120596 in ACSF, 0.5 mM choline was pressure-applied to CA1 pyramidal neurons or CA1 interneurons via a picospritzer pipette every 3 min. Representative traces (A–B) illustrate responses that were obtained from CA1 pyramidal neurons (A) and CA1 interneurons (B) 12, 24 and 45 min (framed time marks) after addition of 5 µM PNU-120596 to ACSF. Responses of CA1 pyramidal neurons (A) and interneurons (B) were analyzed within a time window between the 12^th^ and the 51^st^ min after the start of wash-in phase of PNU-120596. Net charge of current responses from both types of neurons was measured over a 20 s interval from the moment of choline application. Net charge was then normalized to the neuronal capacitance to account for differences in the neuronal size and plotted as a function of time (C). The results are presented as the mean ± S.E.M. Open triangles illustrate the data points obtained from CA1 pyramidal neurons (n = 7); while open circles represent the data points obtained from CA1 interneurons (n = 7). Closed triangles illustrate values obtained by multiplying the pyramidal response net charge (i.e., open triangles) by a factor of 20.

The densities of expression and thus, the normalized net effect of activation (i.e., response net charge per unit of membrane capacitance per unit of time) of native functional α7* nAChRs in CA1 pyramidal neurons and CA1 interneurons were compared in whole-cell patch-clamp experiments in hippocampal slices. To improve the detectability of responses of pyramidal α7* nAChRs to choline, 5 µM PNU-120596 was added to ACSF. In these experiments, 0.5 mM choline was pressure-applied via picospritzer pipettes positioned in the vicinity of recorded CA1 pyramidal neurons or CA1 interneurons (Figure 1A–B). Choline was applied every 3 min during the wash-in phase of 5 µM PNU-120596 added to ACSF. Net charge of responses of pyramidal neurons (Figure 3A) and interneurons (Figure 3B) was measured over a 20 s interval from the moment of choline application between the 12^th^ and the 51^st^ min of the PNU-120596 wash-in. If the quality of recordings declined during this 39 min time window (i.e., 14 individual applications), then, the data were discarded. Net charge from successful recordings was then normalized to (i.e., divided by) the neuronal capacitance to account for differences in neuronal size among tested neurons. The normalized net charge was then plotted as a function of time from the start of PNU-120596 wash-in (Figure 3C). The results of these experiments demonstrate that the density of expression of functional α7* nAChRs in CA1 pyramidal neurons comprises of ∼5% (i.e., ∼1/20) of the density of functional α7* nAChRs expressed in CA1 interneurons (n = 7; Figure 3C). This finding implies that the normalized net effects of activation (i.e., response net charge per unit of membrane capacitance per unit of time) of α7* nAChRs expressed in CA1 pyramidal neurons is ∼20-fold weaker than the net response of activation of functional α7* nAChRs expressed in CA1 interneurons. In these experiments, ACSF contained 20 µM gabazine, 15 µM DNQX, 50 µM AP-5, 40 µM picrotoxin and 0.3 µM TTX. The internal solution contained CsMeSO_3_ (see Methods).

One limitation of this approach is that it does not fully account for potential differences in the distribution of functional α7* nAChRs in CA1 pyramidal neurons and CA1 interneurons. For instance, upon focal pressure application of 0.5 mM choline, dendritic α7* nAChRs (i.e., distal location relative to the application tip) will be exposed to a somewhat lower concentration of choline than somatic α7* nAChRs (i.e., proximal location relative to the application tip) because of the mostly somatic position of the application pipette tip (white arrows, Figure 1A–B). Therefore, in this approach, the contribution of dendritic α7* nAChRs to the net response may have been somewhat underestimated.

### The effects of physiological levels of choline in the presence of PNU-120596

**Figure 4:**
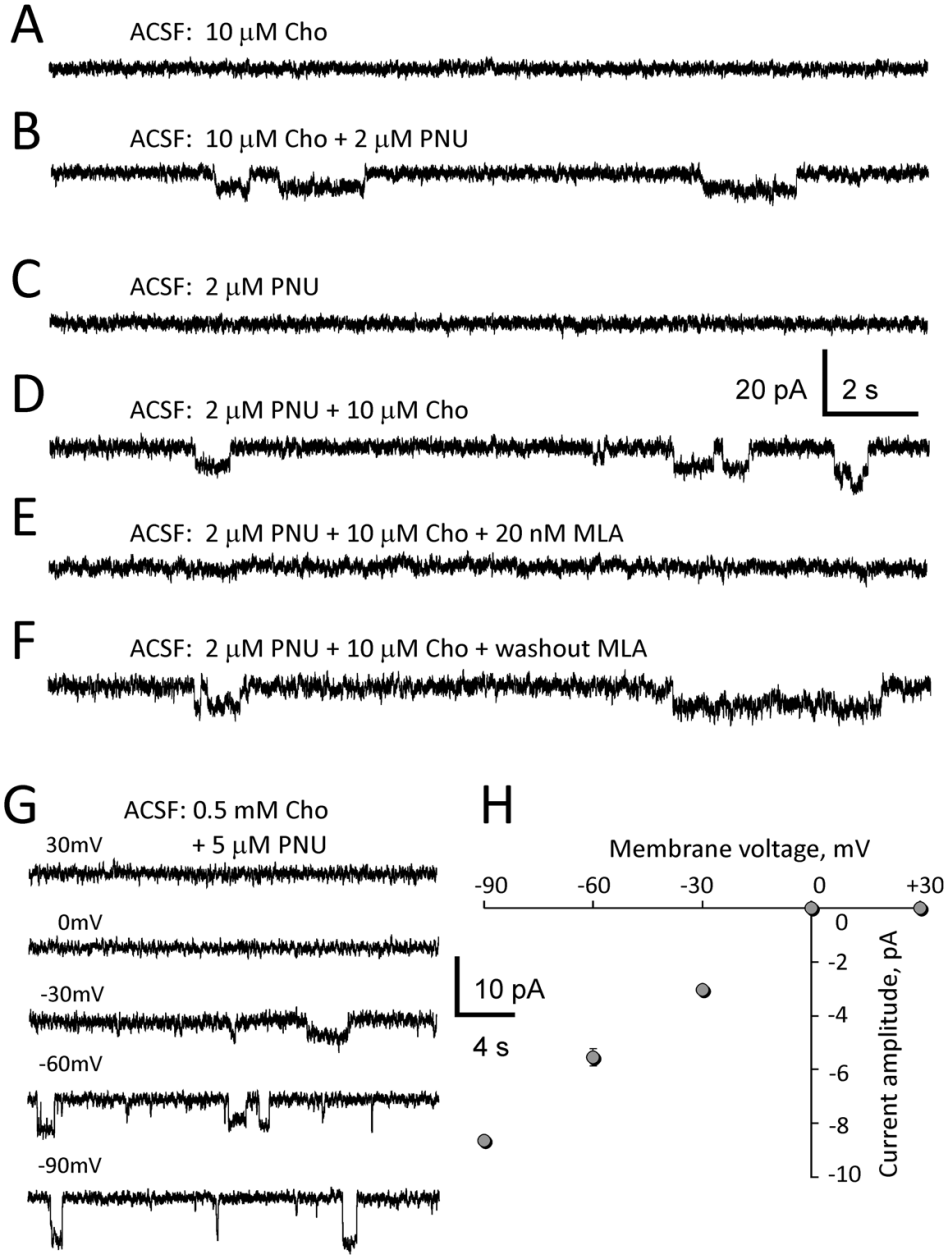
Responses of hippocampal CA1 pyramidal neurons to physiological concentrations of choline in ACSF in the presence of 2 µM PNU-120596. Physiological concentrations of choline alone (i.e., 10 µM; A) or 2 µM PNU-120596 alone (C) added to ACSF, did not elicit responses in CA1 pyramidal neurons in voltage-clamp whole cell experiments. By contrast, addition of 2 µM PNU-120596 to ACSF (containing 10 µM choline) for at least 20 min (B), or addition of 10 µM choline to ACSF (containing 2 µM PNU-120596) for at least 20 min (D) elicited repetitive step-like current deviations completely and reversibly inhibited by 20 nM MLA (E–F). The current-voltage relationship (n = 6) built for step-like current deviations was inwardly rectified and lacked the outward component – a typical property of α7* nAChRs (G–H).

Administration of physiological concentrations of choline (i.e., ∼10 µM) to ACSF is a more adequate model of the effects of endogenous choline and systemic drug administration than focal pressure application of 0.5–1 mM choline. Administration of 10 µM choline alone to ACSF (n = 6; Figure 4A) or 2 µM PNU-120596 alone to ACSF (n = 6; Figure 4C) did not elicit detectable responses in CA1 pyramidal neurons when the drugs were administered for over 1 hr. It is worth mentioning that in one experiment, two step-like current deviations (850 ms and 900 ms in duration) were detected within 1 min from one another after ∼94 min of administration of 2 µM PNU-120596 in the absence of exogenous choline (not shown). These unusual openings were attributed to a potential presence in hippocampal slices of extremely small amounts of endogenous α7* nAChR agonists (e.g., ACh and/or choline). This rare ion channel activity in the absence of exogenous choline was not investigated in this study.

By contrast, the addition of 2 µM PNU-120596 to ACSF containing 10 µM choline (Figure 4B), or the addition of 10 µM choline to ACSF containing 2 µM PNU-120596 (Figure 4D) resulted in repetitive step-like current deviations in 13 out of 16 voltage-clamp whole-cell experiments (∼81%; Table 1). Step-like responses were reminiscent of openings of individual ion channels and were completely and reversibly blocked by 20 nM MLA supporting the involvement of α7* nAChRs (n = 5; Figure 4E–F). The mean duration, the frequency and the mean net charge of step-like current deviations are presented in Table 1. To evaluate net charge, the area under step-like current deviations was measured over a 10 min interval at least 20 min after administration of 2 µM PNU-120596 to ACSF. At the time of application of PNU-120596, 10 µM choline was already present in ACSF for at least 20 min.

**Table 1:** Properties of currents elicited by 10 µM choline plus 2 µM or 5 µM PNU-120596.

ACSF:	# cells	Mean open time (s)	Frequency of step-like current deviations (events/min)	Net charge (pC/min)	Frequency of action potentials (APs/min)
10 µM choline +2 µM PNU-120596	13	1.1±0.7	**1.8±1.7**	**9.3±9.6**	**6.8±6.0** & (n = 7)
10 µM choline +5 µM PNU-120596	10	1.0±0.3	112.2±41.6*	53.4±23.6*	24.5±18.0*

*The results of experiments where 10 µM choline plus either 2 µM (top row) or 5 µM PNU-1205096 (bottom row) were applied to ACSF for at least 20 min and step-like current deviations in voltage-clamp or action potentials in current-clamp were recorded and analyzed. Significant differences were observed among the frequencies (p = 0.0001) and net charge (p = 0.0001) of step-like current deviations, but not the mean open time (p>0.67). Significant differences were also observed between the frequencies of action potentials (p<0.025).*

& *) Note that in the presence of 10 µM choline plus 2 µM PNU-120596, action potentials were observed in only 7 out of 13 CA1 pyramidal neurons.*

A similar protocol was used in 12 experiments where 5 µM PNU-120596 (instead of 2 µM) was added to ACSF containing 10 µM choline and step-like responses were observed in 10 out of 12 voltage-clamp whole-cell experiments (Table 1). In these experiments, the frequency of step-like current deviations and the mean net charge (but not the mean open time of current deviations) were significantly greater than the corresponding values measured in experiments with 2 µM PNU-120596 (Table 1). These results demonstrate that, in the presence of PNU-120596, the enhanced responsiveness of pyramidal α7* nAChRs to choline results from a synergistic action of choline and PNU-120596 and that the potentiation of 10 µM choline-mediated responses by PNU-120596 is concentration dependent. In these experiments, ACSF contained 20 µM gabazine, 15 µM DNQX, 50 µM AP-5, 10 µM atropine, 40 µM picrotoxin and 0.3 µM TTX.

#### The current-voltage relationship

To build the current-voltage relationship of step-like current deviations, 0.5 mM choline plus 5 µM PNU-120596 were added to ACSF and step-like responses were recorded in voltage-clamp at various membrane potentials between −90 mV and +30 mV with a step of 30 mV (n = 6; Figure 4G). The current-voltage relationship was inwardly rectified and lacked the outward current component (n = 6; Figure 4H), a typical feature of α7* nAChRs in the presence of external and internal Mg^2+^ [59]. These results further supported the enhanced sustained activation of CA1 pyramidal α7* nAChRs by 10 µM choline in the presence of PNU-120596.

### The effects of 10 µM choline plus 2–5 µM PNU-120596 on the excitability of CA1 pyramidal neurons

**Figure 5:**
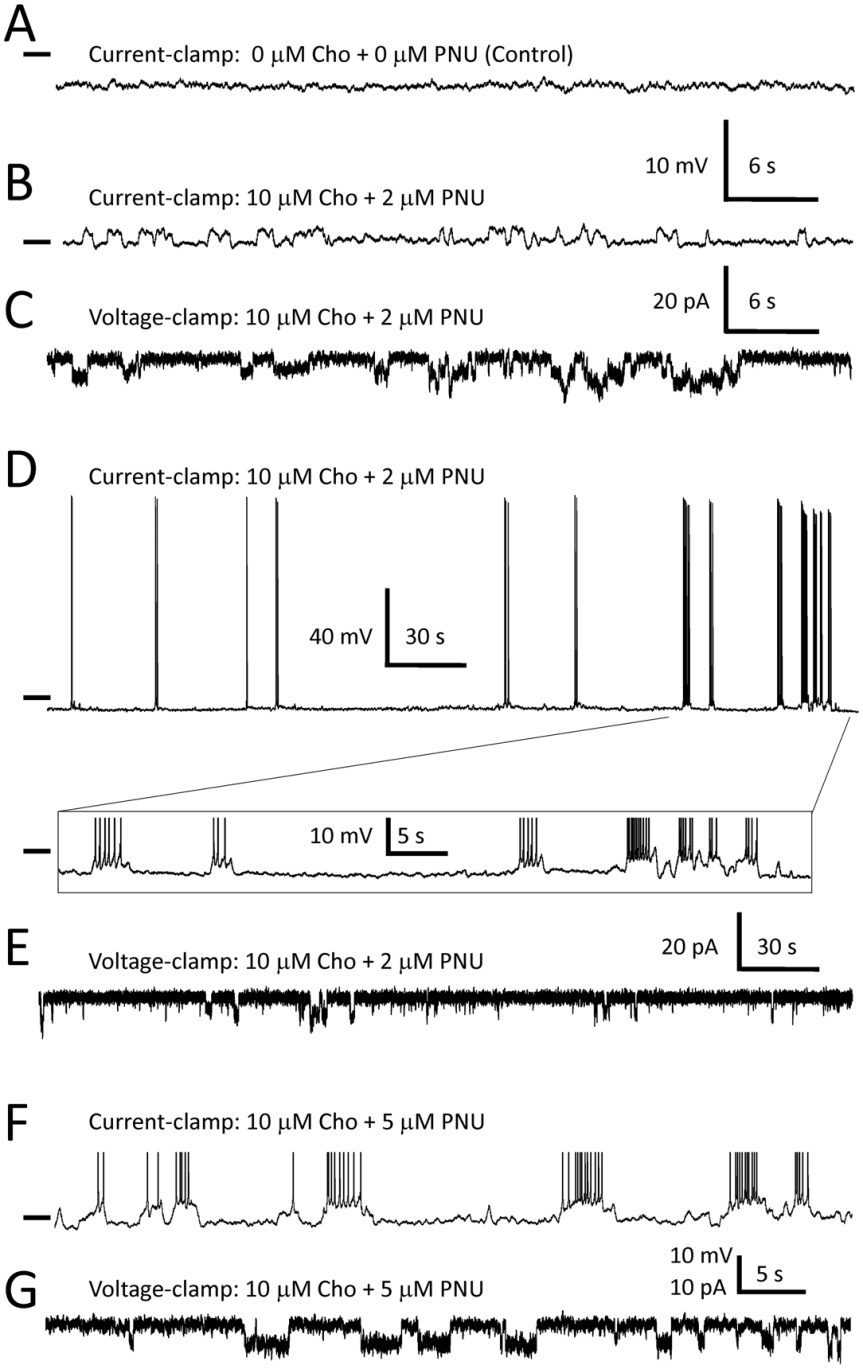
Enhanced excitability of hippocampal CA1 pyramidal neurons in the presence of PNU-120596 and physiological levels of choline. In the absence of choline and PNU-120596, but in the presence of an inhibitory cocktail containing 20 µM gabazine, 15 µM DNQX, 50 µM AP-5, 10 µM atropine and 40 µM picrotoxin, hippocampal CA1 pyramidal neurons did not exhibit any spontaneous activity in current-clamp experiments (A). Addition of 10 µM choline plus 2 µM PNU-120596 to ACSF for at least 20 min elicited step-like voltage deviations in current-clamp (B) and step-like current-deviations in voltage-clamp (C). Traces shown in B and C were recorded from the same CA1 pyramidal cell ∼1 min apart. In some current-clamp experiments, step-like voltage deviations elicited by 10 µM choline plus 2 µM PNU-120596 triggered full action potentials (D). Action potentials shown in F) and in the framed insert to D) were truncated. Traces shown in D and E were obtained from the same cell ∼1 min apart. Similar results were obtained when 10 µM choline plus 5 µM (instead of 2 µM) PNU-120596 were applied to ACSF (F–G and Table 1). Traces shown in F and G were recorded from the same cell ∼1 min apart. Short horizontal bars in front of voltage traces correspond to the membrane voltage of −60 mV.

In the absence of choline and PNU-120596, and in the presence of 20 µM gabazine, 15 µM DNQX, 50 µM AP-5, 40 µM picrotoxin and 10 µM atropine, the mean resting potential of hippocampal CA1 pyramidal neurons was estimated to be −60.0±2.2 mV (n = 64). Under these conditions, CA1 pyramidal neurons were not spontaneously active and did not exhibit step-like voltage deviations during current-clamp recordings (n = 4, Figure 5A). By contrast, upon administration of 10 µM choline plus 2–5 µM PNU-120596 for at least 20 min, step-like voltage (Figure 5B) or current (Figure 5C) deviations were observed in current- or voltage-clamp experiments, respectively. Traces shown in Figures 5B and 5C were obtained from the same CA1 pyramidal neuron in current- and voltage-clamp configurations, respectively, ∼1 min apart.

In current-clamp experiments, in the presence of 10 µM choline plus 2 µM PNU-120596 added to ACSF for at least 20 min, step-like voltage deviations triggered action potentials in α7 out of 24 (∼29%) CA1 pyramidal neurons tested (Figure 5D). In the remaining 17 cells, step-like voltage-deviations did not trigger action potentials (Figure 5B). Traces shown in Figures 5D and 5E were obtained from the same CA1 pyramidal neuron in current- and voltage-clamp configurations, respectively, ∼1 min apart.

When 10 µM choline plus 5 µM PNU-120596 were added to ACSF, action potentials were triggered by step-like voltage deviations in over twice as many CA1 pyramidal neurons: 12 out of 17 (∼70.6%) cells tested (Figure 5F). The average number of action potentials elicited by step-like voltage-deviations was estimated to be 6.8±6.0 APs/min (n = 7) in the presence of 10 µM choline plus 2 µM PNU-120596 and 24.5±18.0 APs/min (n = 10) in the presence of 10 µM choline plus 5 µM PNU-120596 (Table 1) – a statistically significant difference (p<0.025). Traces shown in Figures 5F and 5G were obtained from the same CA1 pyramidal neuron in current- and voltage-clamp configurations, respectively, ∼1 min apart.

These results demonstrate that in the presence of PNU-120596 in ACSF, individual openings of α7* nAChR ion channels appear to be detectable in whole-cell voltage- and current-clamp recordings as step-like current and voltage deviations, respectively. In current-clamp experiments, these putative single channel openings resulted in small repetitive step-like depolarizations that can occasionally excite hippocampal CA1 pyramidal neurons. Therefore, in the presence of PNU-120596, physiological concentrations of choline appear to enhance the excitability of CA1 pyramidal neurons and these effects are concentration-dependent.

### Evaluation of the first latency time and wash-out rates of the effects of choline plus PNU-120596 applied to ACSF

The first latency time (i.e., time between the start of drug application to ACSF and the first step-like current deviation) was 6.8±1.6 min (n = 6) upon addition of 5 µM PNU-120596 to ACSF that contained 0.5 mM choline for at least 30 min. The first latency time was 3.1±0.2 min (n = 4) upon addition of 0.5 mM choline to ACSF that contained 5 µM PNU-120596 for at least 30 min. Therefore, the onset of effects of 0.5 mM choline (in the continued presence of 5 µM PNU-120596) was approximately 2.2-fold faster than the onset of effects of 5 µM PNU-120596 (in the continued presence of 0.5 mM choline). The first latency times likely reflected the method of drug administration employed in this study (drugs were added to ACSF), the speed of ACSF perfusion (1 ml/min), the volume of the recording chamber (∼2 ml), the location of recorded neurons within the slice and the location of slices within the recording chamber. However, the 2.2-fold difference in the first latency times for wash-in of choline and PNU-120596 likely reflected differences in the rates of equilibration of these drugs in hippocampal tissues and was determined by the drug lipophilicity, molecular size, diffusion coefficient, receptor binding properties and other biophysical properties.

**Figure 6:**
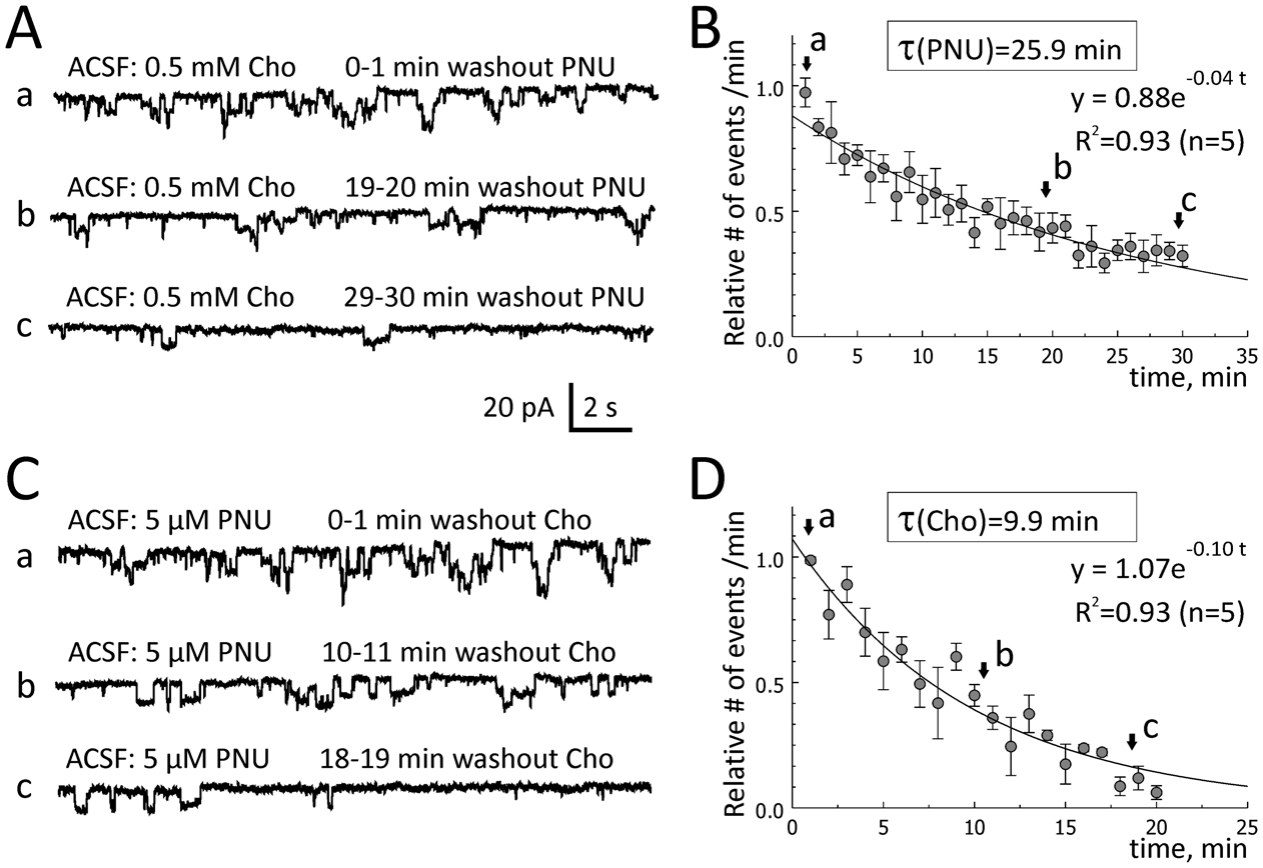
Washout rates of choline and PNU-120596 in hippocampal slices. To evaluate the rates of washout of choline and PNU-120596 in hippocampal slices, 0.5 mM choline and 5 µM PNU-120596 were added to ACSF for ∼1 hr and step-like current deviations were recorded as controls (Aa and Ca). One of the compounds (i.e., choline or PNU-120596) was then removed from ACSF while the other remained. When PNU-120596 was removed from the mix (Ab–Ac), the frequency of step-like current deviations (events/min) dropped exponentially with the time constant, 25.9 min (n = 5, A–B). When choline was removed from the mix (Cb–Cc), the frequency of step-like current deviations also dropped exponentially, but with the time constant, 9.9 min (n = 5, C–D), i.e., ∼2.6 times faster.

To determine the washout rates of choline and PNU-120596, 0.5 mM choline plus 5 µM PNU-120596 were added to ACSF for ∼1 hour to elicit repetitive step-like responses in voltage-clamp experiments (Figures 6Aa and 6Ca). Choline or PNU-120596 was then removed from ACSF one at a time, while the other compound remained in ACSF. As the effects of choline plus PNU-120596 were synergistic (Figure 4A–D), a removal of either of the two components led to a reduction in the frequency of step-like current deviations (Figures 6Ab-c and 6Cb-c). The frequency of step-like responses was measured in events per minute and plotted as a function of time (Figures 6B and 6D). The data were fitted with single exponential functions and the decay time constants were determined. The washout time constant of 5 µM PNU-120596 (in the continued presence of 0.5 mM choline in ACSF) was 25.9 min (n = 5; Figure 6B; an exponential fit (R^2^ = 0.93): y = 0.88exp(−0.04 t); whereas the washout time constant of 0.5 mM choline (in the continued presence of 5 µM PNU-120596 in ACSF) was 9.9 min (n = 5; Figure 6D; an exponential fit (R^2^ = 0.93): y = 1.07exp(−0.10 t), where t is the washout time in minutes. Therefore, the washout of 0.5 mM choline from hippocampal tissue (in the continued presence of 5 µM PNU-120596 in ACSF) was ∼2.6-fold faster than the washout of 5 µM PNU-120596 (in the continuing presence of 0.5 mM choline in ACSF). Thus, the rates of both wash-in and washout of PNU-120596 appear to be considerably slower than the rates of wash-in and washout of choline, respectively. Again, these differences likely reflect differences in the rates of equilibration of these drugs within hippocampal tissue. In these experiments, ACSF always contained 20 µM gabazine, 15 µM DNQX, 50 µM AP-5, 40 µM picrotoxin and 0.3 µM TTX.

## Discussion

Deficits in hippocampal α7* nAChR activation are a key accompanying factor in certain cognitive disorders and enhancing this activation by nicotinic agonists has been shown to produce cognitive benefits. Choline is an endogenous selective α7 nAChR agonist but present in the cerebrospinal fluid (CSF) at much lower concentrations (∼10 µM) relative to its EC_50_ (i.e., ∼0.5–1.5 mM; [60], [61]). Therefore, endogenous choline may not be effective in activation of native hippocampal CA1 pyramidal α7* nAChRs in the absence of positive allosteric modulators, such as PNU-120596. In fact, this is true even for hypothalamic tuberomammillary neurons that express much higher densities of α7* nAChRs [43] than CA1 pyramidal neurons. This conclusion also complements previous reports demonstrating that choline exhibits a greater potency for desensitization (IC_50_ ∼40 µM, [56]) than activation of α7* nAChRs (EC_50_ ∼0.5–1.5 mM; [60], [61]). PNU-120596 reduces desensitization of α7 nAChRs and thus, increases the potency of α7 nAChR agonists including choline. Accordingly, this study tested and confirmed the hypothesis that in the presence of PNU-120596, physiological levels of choline (∼10 µM) become effective in activation of native functional α7* nAChRs expressed in hippocampal CA1 pyramidal neurons in relatively low densities. In the presence of PNU-120596, physiological concentrations of choline were found to elicit a weak persistent activation of CA1 pyramidal α7* nAChRs and enhance the excitability of CA1 pyramidal neurons – effects that in *in vivo* settings may produce neuroprotection [14], [22], [62], [63] and cognitive benefits [11], [64].

In the initial series of experiments, focal pressure application of 0.5–1 mM choline was used to demonstrate that: 1) The majority of hippocampal CA1 pyramidal neurons express functional α7* nAChRs; 2) A lack of responsiveness of some hippocampal CA1 pyramidal neurons to focal application of 0.5–1 mM choline in the absence of PNU-120596 does not imply a lack of expression of functional α7* nAChRs in these neurons, but may indicate a lack of detection of α7* nAChR-mediated currents by patch-clamp electrophysiology; and 3) PNU-120596 can serve as a powerful tool for detection of low level expression and activation of functional α7* nAChRs such as those expressed in hippocampal CA1 pyramidal neurons.

To model the effects of endogenous choline on CA1 pyramidal α7* nAChRs and neurons, physiological concentrations of choline (i.e., ∼10 µM) were added to ACSF and whole-cell voltage- and current-clamp recordings were conducted in the presence and absence of 2–5 µM PNU-120596 (Figures 4–5). These experiments revealed that in the presence of PNU-120596 and 10 µM choline, low densities of expression of CA1 pyramidal α7* nAChRs generate persistent step-like currents which cause transient depolarizations and occasionally, may trigger bursts of action potentials (Figure 5). It is this capability of as few as only one individual functional α7* nAChR to depolarize and excite the entire neuron that makes it possible for a low density expression of functional α7* nAChRs to be effective in enhancing the excitability of hippocampal CA1 pyramidal neurons in the presence of PNU-120596. Therefore, high levels of expression of CA1 pyramidal α7* nAChRs and synchronization of their activity are not required for significant depolarizing and excitatory effects of physiological concentrations of choline plus PNU-120596 on CA1 pyramidal neurons. This may be important because deficits in activation of hippocampal α7* nAChRs have been linked to certain cognitive disorders and enhancing α7* nAChR activation by systemic administration of nicotinic agonists has been shown to produce neuroprotection and cognitive benefits [15], [16], [17], [18], [19], [21], [28], [29], [30], [57], [65]. For example, a weak but persistent activation of α7* nAChRs can be achieved with low concentrations of nicotinic agonists (e.g., ∼1–6 µM 4OH-GTS-21 (i.e., 3-(4-hydroxy, 2-methoxy-benzylidene)anabaseine), a selective α7 nAChR agonist [56], [66]) and has been shown to be neuroprotective in the NGF/serum-withdrawal toxicity model in pheochromocytoma-12 cells expressing functional α7* nAChRs [22]. Therefore, the results of this study suggest that in the presence of PNU-120596, endogenous choline may produce a weak but persistent activation of functional α7* nAChRs in hippocampal CA1 pyramidal neurons and thus, may act as a potent therapeutic agent with potential neuroprotective and cognition-enhancing properties.

In the absence of PNU-120596, the mean open time of hippocampal α7* nAChRs is very short (∼100 µs, [54]) and therefore, only a highly synchronized activity of α7* nAChRs can be confidently detected in voltage-clamp whole-cell experiments. Indeed, in this study, fairly synchronous, although small, responses were occasionally observed when high concentrations of choline (0.5–1 mM) were focally applied via a picospritzer pipette to recorded CA1 pyramidal neurons (Figure 1D). A lack of substantial delay between pressure applications of choline and whole-cell responses of CA1 pyramidal neurons suggests that at least some pyramidal α7* nAChRs were expressed somatically (Figures 1D–E and 2E). However, when considerably lower concentrations of choline (e.g., 10 µM) were administered to ACSF in the absence of PNU-120596, responses of pyramidal α7* nAChRs were not detectable in whole-cell experiments (Figure 4A). By contrast, in the presence of PNU-120596, 10 µM choline applied to ACSF elicited clearly detectable responses (Figures 4B and 4D). These effects are directly attributable to the synergistic action of 10 µM choline and 2 µM PNU-120596 allowing sub-threshold concentrations of choline to activate α7* nAChRs [43]. The mean open time of current deviations elicited by 10 µM choline in the presence of 2–5 µM PNU-120596 was estimated to be ∼1.0 s (Table 1). Therefore, in the presence of PNU-120596, a weak α7* nAChR activity has a better chance of being detected in voltage- or current-clamp whole-cell experiments than in the absence of PNU-120596, and the synchronization of α7* nAChR activity is not necessary for detection. Indeed, co-administration of 10 µM choline and 2–5 µM PNU-120596 elicited clearly detectable step-like whole-cell responses in voltage- and current-clamp experiments (Figures 4 and 5). These responses were completely and reversibly blocked by 20 nM MLA supporting the activation of α7* nAChRs (Figure 4E–F).

Intriguingly, current and voltage deviations recorded in voltage- and current-clamp, respectively, in the presence of 10 µM choline plus 2–5 µM PNU-120596 were step-like and thus, reminiscent of single ion channel openings similar to step-like responses observed previously in hypothalamic tuberomammillary neurons which in contrast to hippocampal CA1 pyramidal neurons express very high densities of functional α7* nAChRs [43]. Therefore, it is likely that step-like responses elicited by synergistic action of 10 µM choline and 2–5 µM PNU-120596 result from openings of individual α7* nAChR ion channels detectable in whole-cell patch-clamp experiments.

Detecting activity of individual α7* nAChR ion channels in whole-cell patch-clamp experiments appears to be possible if the probability of ion channel openings is sufficiently low; and if the channels remain open for a prolonged period of time during which the ionic gradient across the membrane and thus, the ionic current, remain relatively constant. These requirements appear to be fulfilled for α7* nAChRs activated by physiological concentrations of choline in the presence of 1–5 µM PNU-120596 in hippocampal CA1 pyramidal neurons (this study), hippocampal CA1 interneurons (Kalappa and Uteshev, unpublished observations) and hypothalamic TM α7* nAChRs [43]. In fact, the probability of openings (P_open_) of α7* nAChRs expressed in TM neurons has been estimated to be so low that in the presence of 10 µM choline plus 1 µM PNU-120596, only N_TM_P_open_∼0.27 α7* nAChR ion channels appeared to be open in the entire TM neuron at any given time [43], where N_TM_ is the total number of detectable α7* nAChRs in a TM neuron.

In this study, the mean amplitude of current deviations and the mean net charge per min generated by pyramidal α7* nAChR ion channels in response to 10 µM choline plus 2 µM PNU-120596 were estimated to be ∼5.5 pA (Figure 4H) and ∼9.3 pC/min = 0.16 pA (Table 1). Therefore, under these conditions, the mean number of α7* nAChR ion channels opened in the entire hippocampal CA1 pyramidal neuron at any given time would be estimated to be N_pyr_P_open_∼0.029 ( = 0.16pA/5.5pA), where N_pyr_ is the total number of detectable α7* nAChRs in a pyramidal neuron. Note that in experiments with TM neurons, 10 µM choline plus 1 µM PNU-120596 were used [43], whereas in this study, the concentration of PNU-120596 was increased to 2 µM because of the substantially lower levels of expression of functional α7* nAChRs in hippocampal CA1 pyramidal neurons compared to TM neurons. However, the exact subunit compositions of functional α7* nAChRs expressed in CA1 pyramidal and TM neurons remain unknown and may be different.

Native α7* nAChRs are highly permeable to Ca^2+^ ions with the permeability ratio P_Ca_/P_Na_ ∼6.0 [67], [68]. This value translates into the fractional Ca^2+^ current, P_f_(α7*)∼10%, at −60 mV [68]. Therefore, the persistent activation of CA1 pyramidal α7* nAChRs in the presence of 10 µM choline plus 2 µM PNU-120596 would be expected to result in a persistent influx of Ca^2+^ ions at a rate of ∼0.016 pA (i.e., 10% of 0.16 pA). Although small, this persistent Ca^2+^ current may be physiologically relevant and support neuroprotection [14], [16], [21]. Moreover, if injected in a small volume of a pre-synaptic terminal expressing α7* nAChRs, it may prime the terminal for plastic changes and thus, may assist in modulation of neurotransmitter release. However, a possibility that this relatively low, but persistent, rate of Ca^2+^ influx is cytotoxic cannot be presently ruled out.

One limitation of this study is that only relatively short-term effects (<3 h) were investigated. Prolonged exposure of CA1 pyramidal α7* nAChRs to choline and PNU-120596 may cause neurotoxicity due to a decrease in receptor desensitization and the high Ca^2+^ permeability of α7* nAChRs. Another limitation is that all experiments in this study were conducted at room temperature and the results may be somewhat altered by physiological temperatures. One additional limitation comes from the fact that in this study pyramidal neurons were pharmacologically isolated and the majority of inhibitory (glycine/GABA_A_) and excitatory (AMPA/NMDA) inputs were blocked by an inhibitory cocktail (see Methods). Finally, the number of step-like current deviations may have been underestimated because not all openings of individual α7* nAChR ion channels may have been detectable in whole-cell experiments. For example, responses of α7* nAChRs located in distal dendritic regions may not have been detected even in the presence of PNU-120596, due to electrotonic filtering. As a result, the estimated value of N_pyr_P_open_ may have been somewhat underestimated.

Hippocampal CA1 pyramidal neurons directly contribute to generation of the hippocampal output and express two predominant types of pre- and post-synaptic functional nAChRs: α7- and α4β2-containing. The location and timely activation of these receptors by pressure-applied ACh have been shown to modulate and determine the sign of synaptic plasticity and the hippocampal output [38], [69]. CA1 pyramidal neurons receive inhibitory GABAergic inputs from CA1 interneurons that express high densities of α7* nAChRs (Figure 3B–C; [33], [35], [70]). CA1 interneurons may directly inhibit CA1 pyramidal neurons via GABAergic synaptic inputs, or may excite CA1 pyramidal neurons by inhibiting other CA1 interneurons (i.e., via disinhibition) [69]. Although PNU-120596 would be expected to enhance activation of α7* nAChRs in pyramidal neurons and interneurons proportionally (Figure 3), the net effect of this activation remains unclear and is likely to be concentration-dependent. Moreover, in the presence of PNU-120596, the activation of α7* nAChRs by ACh would be expected to be substantially enhanced, while the activation of non-α7* nAChRs should remain unchanged. Therefore, in the presence of PNU-120596, the net effect on the hippocampal output of activation of CA1 α7* nAChRs will likely depend on the strength, timing and location of cholinergic terminals and the relative densities of expression of pre- and postsynaptic α7* and non-α7* subtypes of nAChRs, as discussed previously [38], [69]. In the present study, the net effect of activation of CA1 α7* nAChRs was not investigated, as the majority of excitatory and inhibitory inputs to pyramidal neurons were blocked by an inhibitory cocktail (see Methods) and the effects of activation of muscarinic AChRs were eliminated by using choline and atropine. However, future studies may use the presented in this study protocols to answer an important question as to how a concurrent activation of CA1 pyramidal neurons and CA1 interneurons by physiological concentrations of choline and 1–5 µM PNU-120596 affects the excitability of pyramidal neurons and thus, the hippocampal output.

Although hippocampal CA1 pyramidal neurons express functional α7* nAChRs, it remains to be determined whether cholinergic terminals from the basal forebrain directly innervate these neurons by establishing functional cholinergic α7* nAChR-containing synapses, or activate pyramidal α7* nAChRs indirectly, i.e., via volume transmission. It is also unknown whether endogenously released ACh can reach pyramidal α7* nAChRs at concentrations sufficient for generating physiologically significant effects in the absence or presence of PNU-120596. Nevertheless, the presented results support the hypothesis that despite low densities of expression of functional α7* nAChRs in hippocampal CA1 pyramidal neurons, in the presence of PNU-120596 endogenous choline may persistently activate these receptors and enhance the excitability of hippocampal CA1 pyramidal neurons in the absence of exogenous nicotinic agents and endogenously released ACh. Therefore, in the presence of PNU-120596, endogenous choline may serve as an efficacious therapeutic agent in cholinergic therapies aimed at recovering the deficiency in hippocampal α7* nAChR activation. Treatments involving endogenous choline may also be safer than those involving exogenous α7* nAChR agonists.

The expression of functional α7* nAChRs in brain tissues can be estimated by [^125^I] α-bungarotoxin binding, a selective α7* nAChR antagonist [71]. The results of [^125^I] α-bungarotoxin binding in the hippocampal CA1 pyramidal region demonstrated that the level of expression of α7* nAChRs is age-dependent [71], peaks on postnatal days 2–5 (i.e., P2–5) and then declines to the adult level by P20. In the present study, the majority of experiments were conducted using P22–26 rats, therefore the expression of functional hippocampal α7* nAChRs was near its young adult levels [71]. Interestingly, only ∼10% of hippocampal α7 proteins are surface-expressed [32] and therefore, the CA1 hippocampal region may contain a large pool of unused α7 proteins. It is intriguing to speculate that under certain endogenous conditions this pool of dormant α7 proteins can be recruited to become functional and cell surface-expressed. It is also feasible that certain endogenous compounds are capable of enhancing α7* nAChR function in a manner similar to PNU-120596. Finding these endogenous conditions and mechanisms of regulation of α7* nAChR surface expression and function in the hippocampus and other brain regions may have a tremendously positive impact on the future of cholinergic therapies aimed at restoring and boosting cognitive performance.
